# A proposed method to minimize acute kidney injury by avoiding vasopressors during surgery

**DOI:** 10.1080/0886022X.2022.2141647

**Published:** 2022-11-11

**Authors:** Donald H. Lambert

**Affiliations:** Anesthesiology, Boston Medical Center, Boston University School of Medicine Boston MA, USA

To the Editor,

The association of intraoperative vasopressor utilization with acute kidney injury (AKI) reported by Ariyaratna et al. [1] is noteworthy and it suggests an opportunity for anesthesiologists to minimize this injury. Although, the article shows an association of AKI with vasopressor use, it is unclear what causes AKI during surgery. The article raises important questions:

1. Because alpha adrenergic agonists indiscriminately cause vasoconstriction, did their use cause renal ischemia and the resultant AKI?
2. Is the use of vasopressors merely a marker for hypotension, which is the cause of the AKI?
3. Is the combination of hypotension and vasopressor induced ischemia the cause of the AKI?

Hypotension and vasopressor use during surgery has many causes, including hypovolemia, preexisting cardiovascular disease, and aging. Excluding these causes, intraoperative hypotension requiring treatment with vasopressors can be caused by excessive anesthesia [2]. The article suggests that anesthesiologists compensate for hypotension due to deep anesthesia with vasopressor boluses and infusions. This occurs because the goals of anesthesia include providing insensibility to pain, hemodynamic stability, and avoidance of awareness. To avoid awareness anesthesiologists literally guess at how much anesthesia to deliver erring on the side of too much as opposed to too little. One method is to deliver an inhaled anesthetic at a minimum alveolar concentration (MAC), usually 0.7, that assures an absence of awareness [3]. However, in addition to inhaled anesthetics, anesthesiologist also administer a variety of additional medication, e.g. narcotics for analgesia, and benzodiazepines for sedation and amnesia. These additional medications, when added to a MAC of 0.7, frequently cause hypotension that is treated with vasopressors. An alternative is to titrate the proper amount of anesthesia by monitoring the depth of anesthesia with a processed EEG monitor (PEEGM). In this way, hypotension and its treatment with vasopressors can both be avoided, hopefully decreasing the incidence of AKI and other organ damage.

The ‘Proper Anesthetic Depth’ (PAD) is a concept, which attempts to avoid hypotension and vasopressor use. Instead of, relying on a predetermined MAC, which can result in hypotension causing AKI, anesthesiologists need a measure of PAD to judge the intensity of anesthesia that eliminates guesswork. Unfortunately, that measurement does not exist. Until such a measurement is available, a surrogate concept is suggested that utilizes a PEEGM and the mean arterial pressure (MAP) to better judge the depth of anesthesia.

**Figure 1.**
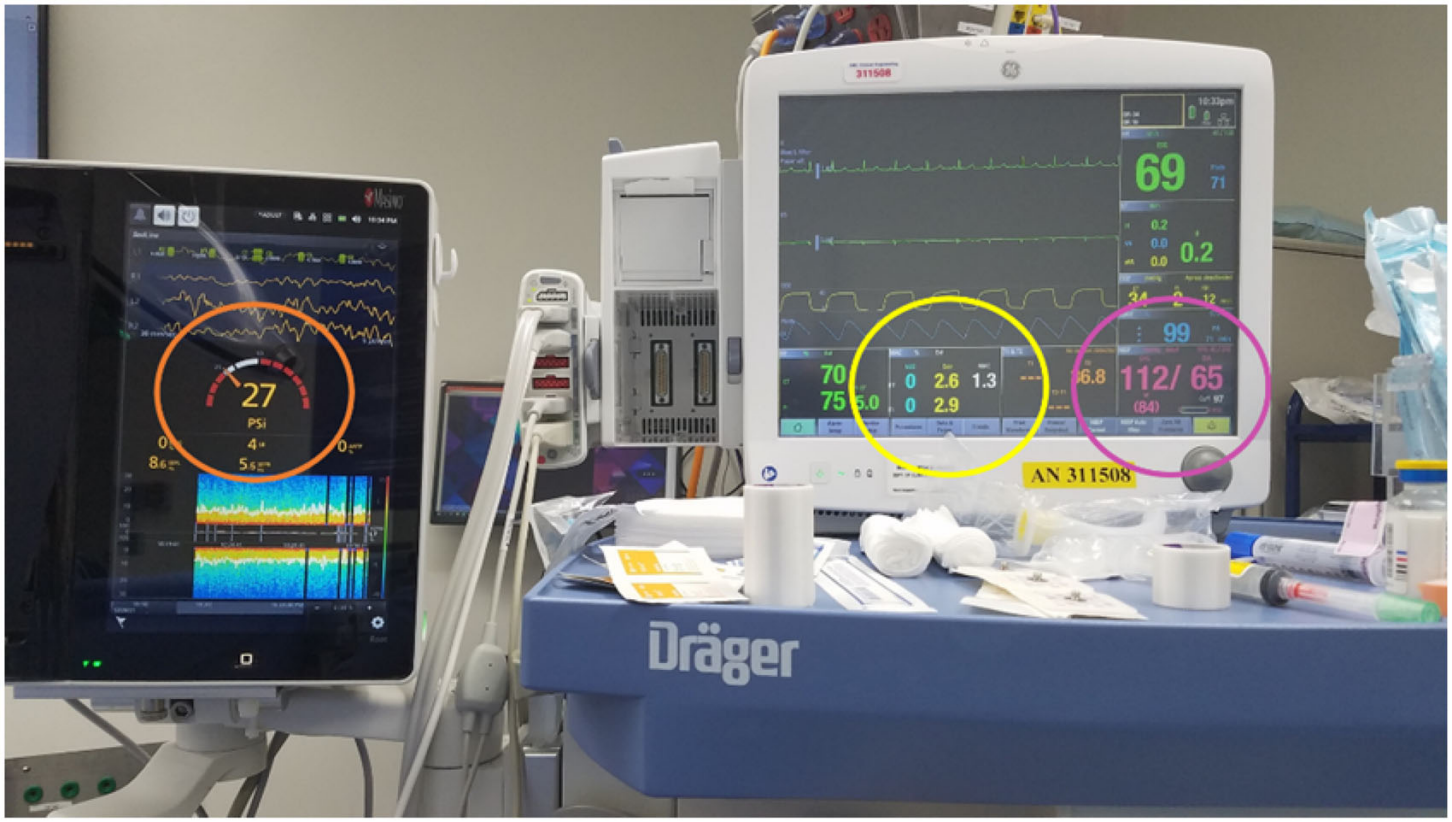
The PAD concept of dependent and independent variables. On the left is the PEEGM, a dependent variable, showing a PSi (patient state indicator) of 27 in the orange circle (normal range 25–50) indicating that the patient is adequately anesthetized. On the right is the blood pressure, a dependent variable showing a MAP of 84 (lavender circle) indicating normotension. In the center (yellow circle) is the independent variable (inhaled and exhaled sevoflurane concentration) showing a MAC of 1.3. The sevoflurane is increased or decreased in order to keep the PEEGM and MAP in the desirable range (65–100 mmHg). The PAD concept, of monitoring the PEEGM and the MAP takes the guesswork out of judging anesthetic depth, thereby preventing the delivery of too little or too much anesthesia, which in turn can cause hypotension and the need for vasopressors.

**Figure 2.**
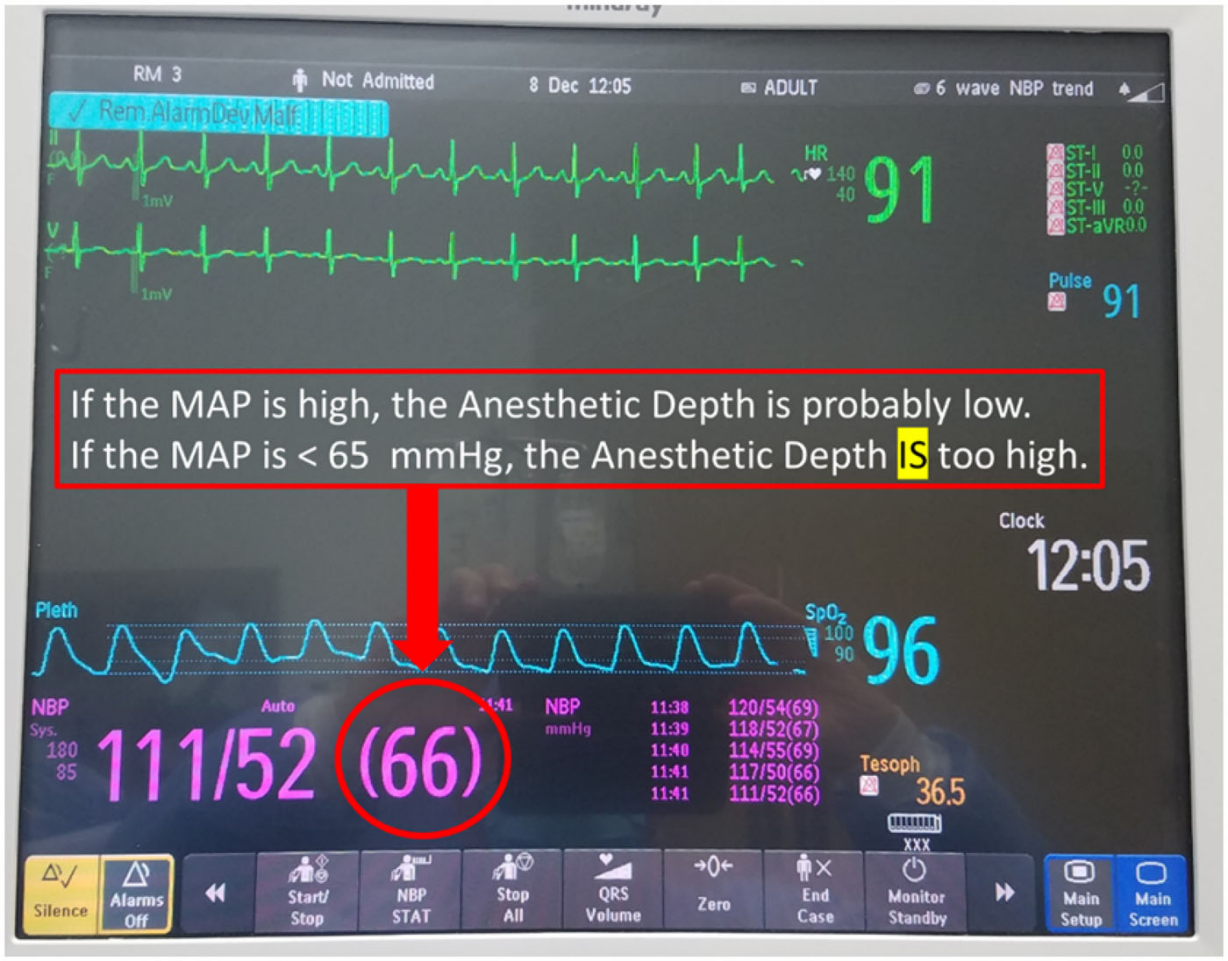
With the PEEGM in the range that indicates lack of awareness, the MAP is used as a guide to the depth of anesthesia, which is controlled by increasing or decreasing the sevoflurane concentration. When the MAP is high, the depth of anesthesia is probably low and indicates the need for more sevoflurane. On the other hand, a MAP < 65 mmHg, is always too low to prevent AKI and indicates a need to decrease the administered sevoflurane.

The PAD concept represents the anesthesia provided by all of the drugs given (inhalational and intravenous). It is illustrated in Figures 1 and 2. In this model, the PEEGM and the MAP are dependent variables, and the inhaled anesthetic concentration is the independent variable that can be increased or decreased to maintain the PEEGM and MAP in a desirable range.

When the PEEGM indicates a lack of awareness, a high MAP (e.g. >100 mmHg) indicates that the depth of anesthesia is probably low, and it is treated by increasing the concentration of the inhaled anesthetic agent. On the other hand, a MAP < 65 mmHg is too low to prevent kidney injury suggesting that the anesthetic depth is unnecessarily high and should be treated by decreasing the inhaled anesthetic concentration. By contrast, a MAP of 70–100 mmHg, which some anesthesiologists might associate with inadequate anesthesia and possible awareness, is not necessarily inconsistent with a PAD. A MAP in the normotensive or even hypertensive range is not associated with AKI or other adverse effects [4]. When a MAP > 65 mmHg is difficult to maintain, the addition of a vasopressor is indicated and should not be avoided [5]. On the other hand, the need for a vasopressor infusion to maintain normotension is an indication that too much anesthesia is being delivered [2].

Utilization of the PAD requires constant ‘vigilance’ (the motto of the American Society of Anesthesiology). It is much easier to produce a very deep anesthetic (typically associated with hypotension) to avoid awareness and maintain a normal MAP with a vasopressor infusion, which we now know is associated with AKI.

The PAD is especially important and most useful between the induction of anesthesia and incision when hypotension is most likely to occur owing to the necessity of providing enough anesthesia to prevent awareness and movement in the absence of surgical stimulation [6]. As suggested by Ariyaratna et al. [1] efforts to avoid perioperative hypotension and vasopressor use may minimize the risk of AKI.

Excluding cardiac and obstetrical anesthesia, which the author does not do, the author utilizes the PAD in all anesthetics, including emergency, and laparoscopic procedures. Although the author does not perform cardiac and obstetrical anesthesia, it is reasonable to expect the PAD will function equally well during cardiac and obstetrical operations.

In conclusion, the PAD is a method that accomplishes the anesthetic goals of avoiding both awareness and hypotension without vasopressors that might prevent AKI. To that end, the author has utilized the PAD for approximately one year and has only rarely resorted to vasopressors to maintain a lack of awareness and adequate MAP.
